# Failure of Digital Device Performance in Monitoring Physical Exercise in a Pilot Study in Sedentary Persons with HIV

**DOI:** 10.3390/s23239461

**Published:** 2023-11-28

**Authors:** Matteo Bonato, Federica Marmondi, Filippo Turrini, Andrea Albergoni, Maddalena Pennacchi, Camilla Cerizza, Maria Francesca Piacentini, Antonella Castagna, Laura Galli, Francesco Sartor, Paola Cinque

**Affiliations:** 1 Department of Biomedical Sciences for Health, Università degli Studi di Milano, 20133 Milan, Italy; 2 IRCCS Istituto Ortopedico Galeazzi, Laboratory of Movement and Sport Sciences (LaMSS), 20157 Milan, Italy; 3 Department of Infectious Diseases, IRCCS San Raffaele Scientific Institute, 20127 Milan, Italy; 4 Department of Neuroscience Rehabilitation, Ophtalmology, Genetics and Maternal Child Health, Università degli Studi di Genova, 16148 Genua, Italy; 5 Department of Movement, Human and Health Sciences, University of Rome ‘Foro Italico’, 00135 Rome, Italy; 6 Department of Patient Care & Monitoring, Philips Research, 5656 AE Eindhoven, The Netherlands; 7 School of Sport, Healthand Exercise Sciences, University of Wales, Bangor L57 2EF, UK

**Keywords:** health, lifestyle, monitoring, physical activity, smart devices

## Abstract

Digital devices have gained popularity in the last 10 years as a tool for exercise prescription, the monitoring of daily physical activity, and nutrition for the management of a health-related parameter. Therefore, the aim of this study was to assess the effectiveness of the use of digital devices to monitor exercise data in sedentary persons with HIV who exercise following an individualized activity pacing (AP) protocol on cardiorespiratory fitness body composition, blood lipid profile, and psychological parameters. Twenty-four PLWH were enrolled in an 18-week randomized, open-label, pilot AP exercise protocol. All participants were monitored by a Health Band connected to a mobile app that transmitted the data to a server. At week 3, they were randomized either in an experimental group (EG), in which an open device configuration enabled them to receive training data feedback (*n* = 12), or continued with no data feedback (control group, *n* = 12). The primary endpoint was improvement from the baseline of 15% of steady-state oxygen consumption ($\dot{V}$O_2_) during a 6-min walking test. Technical issues occurred when pairing the health band with the app, which prevented EG participants from regularly receiving data feedback, and with data transmission to the server, which enabled only 40% monitoring of the total training days. Consequently, the study outcomes could not be compared between the two groups, and participants also lost confidence in the study. However, 19 out of 24 participants completed the AP program. Overall, only 6 (32%) improved steady-state $\dot{V}$O_2_, with no significant changes at W18 from the baseline. Significant reductions were observed of BMI (*p* = 0.040), hip circumference (*p* = 0.027), and total-(*p* = 0.049) and HDL-cholesterol (*p* = 0.045). The failure of digital device performance substantially affected study procedures, monitoring, and participants’ engagement, and likely limited the potential benefits of the AP exercise program.

## 1. Introduction

In the last years, wearable technology or applications on smartphones or tablets have gained popularity for monitoring physical activity and health measures, now representing a popular trend in health and fitness [1]. Such smart technology is useful to support physical activity by providing motivational input through feedback and personalized coaching. Digital devices may help track physical activity through several measures, such as the number of steps, walked or run distance, sedentary time, energy expenditure, or heart rate (HR) [2]. Furthermore, they can facilitate self-monitoring and goal setting, and thus motivate and reinforce positive behavior for physical activity participation [3,4].

Because of their potential to collect activity, fitness, and other health-related parameters, digital devices can be particularly useful for exercise interventional prospective studies of clinical and non-clinical populations. In this context, they will provide a more precise approach for data collection than using self-reported questionnaires and diaries, which may suffer from participants’ biases due to social desirability or imprecise recall [5,6]. Accurate data collection is also of relevance for monitoring adherence to exercise. However, poor information is available regarding the actual potential of digital devices in promoting adherence to exercise.

In persons with human immunodeficiency virus (HIV), the use of digital devices has been shown to improve physical activity levels and, consequently, several health outcomes [7]. For instance, these tools have been useful in persons who followed a walking program to collect data on HR and time and distance walked, which enabled them to describe the volume and intensity of physical activity [8]. However, less is known regarding the use of digital devices and their potential to promote exercise execution in persons with HIV. Recently, we also focused on adherence in a 16-week pilot study in persons with HIV who exercise with the support of an ad-hoc smartphone application, as compared to a control group exercising without an application [9]. We observed that only the participants who exercised with the smartphone application improved cardiorespiratory fitness, body composition, cholesterol profile, and psychological outcomes, which supports the usefulness of these devices for training motivation. However, training adherence was unexpectedly similar in both groups, i.e., 60% in the application group (based on the application records) and 54% in the control group (self-reported), which was consistent with an overreport of the frequency and duration of training sessions from the control participants who exercised without an application. These observations indicate the importance of objective measures of adherence.

To assess whether digital instruments may help improve adherence through feedback of daily activities, we designed a pilot control study in sedentary persons with HIV. We prescribed a physical activity program based on activity pacing (AP), a strategy that splits daily activities into smaller and more manageable portions, thus favoring better adherence and a gradual but progressive increase in physical fitness [10]. We expected to observe higher adherence and more health-related quality of life benefits in participants randomized to receive the real time details of their activity (e.g., steps and heart rate) through the smartphone application, compared to participants who did not receive such feedback.

## 2. Materials and Methods

### 2.1. Study Design

In this randomized, open-label, pilot study were enrolled persons with HIV receiving combination antiretroviral therapy (cART). The inclusion criteria included age ≥18 years and having a sedentary lifestyle, defined as less than 2 days of physical activity per week for less than 20 min per session [6]. The exclusion criteria included any disease requiring hospitalization in the 6 weeks before the beginning of the study or any medical conditions (e.g., cardiovascular diseases, musculoskeletal injuries, neurologic disorders) that contraindicated exercise, as established by a sports medicine specialist, current substance abuse, and alcohol abuse.

The protocol had a duration of 18 weeks, divided into a two-week observational period (W0–W2), aimed at collecting useful data to set up the 16-week AP protocol (W3–W18). During W0–W2, participants were monitored daily using a health band (Health Band, Philips Respironics, Pittsburgh, PA, USA) to record steps, activity time, walked distance, HR, and energy expenditure through an actigraphy and heart rate device. The health band provided health data tracking via Bluetooth technology to a mobile application installed on the participants’ smartphones, which were connected to the Philips Actigraphy Server System (PASS) website to monitor the participants’ data. At the end of W2, participants were randomized in a 1:1 ratio to either the experimental (EG) or control group (CG). In both groups, participants used the health band and mobile application; however, the EG participants could monitor exercise intensity and physical activity execution during the training session and received feedback of their training data through the app, whereas the CG participants trained with a blind configuration of the health band and mobile application and received no data feedback. The training data were also automatically downloaded from all participants to the PASS website to be monitored by the study coaches.

The primary objective of the study was the comparison of the proportion of participants with an improvement from the baseline of 15% of steady-state oxygen consumption ($\dot{V}$O_2_) during 6MWT between the two groups. As secondary objectives, we compared changes from the baseline in body composition, blood lipid profile, and psychological outcomes.

The study protocol was approved by the Ethical Committee of San Raffaele Hospital in accordance with the Declaration of Helsinki III. This study was registered at https://clinicaltrials.gov/ (accessed on 28 October 2022, NCT05194059). All study participants signed an informed consent form before the beginning of the study.

### 2.2. Participant Screening and Training Protocol

Participants were enrolled from the outpatient clinic of the unit of Infectious Diseases of the IRCCS San Raffaele Scientific Institute (Milan, Italy), following indications of the caring physicians and using patient communities’ channels. Potential participants were screened by an infectious disease specialist (PC). Moreover, a sports medicine doctor (CC) performed a 12-derivation electrocardiogram (ECG) at rest. Patients who met the above inclusion criteria were enrolled in the study.

The physical activity protocol for EG and CG was designed using an AP strategy, which helped participants manage fatigue by splitting the exercise session into more feasible exercise portions. Following the first two weeks, each participant received a tailored program consisting of 2 to 4 outdoor sessions per week of aerobic training (brisk walking). Professional coaches (FM and MP) prescribed both continuous and intermittent training sessions [11]. Continuous training sessions had a volume that varied among participants from 15 to 45 min, and participants were instructed to manage their physical activity during the day to reach the target. Intermittent training sessions had a volume varying from 15 to 30 min, in which participants alternated bouts at high intensity with passive short recovery periods (e.g., 3 reps of 5 min of brisk walking with 3 min of passive recovery). Each intermittent training session included a 5-min warm-up before and 5-min cool-down at the end. The training intensity for EG participants was set between 60 and 70% of the maximal HR (HR_max_) [12], and between 70 and 80% of HR_max_ for intermittent training. For CG participants and for all participants treated with beta-blockers, the training intensity was set according to the Borg CR-10 category ratio scale to determine the rating of perceived exertion (RPE) from 0.3 to 3 arbitrary unit (A.U.) for continuous training and from 3 to 5 A.U. for intermittent training [13]. Table 1 shows the physical activity progression through all 16 weeks of training for both continuous and intermittent training.

Using PASS log-in credentials, the authorized team members (FM and MP) could access the participants’ data on exercise (identified by a code number) on a customized form. Every week, participants from both EG and CG were contacted by telephone to receive advice on how to optimally manage subsequent sessions (at a perceived fatigue level of 2–3 of the Borg scale) and be counseled about the use of digital devices (by FM and MP). Anonymized data from the PASS server were also transferred onto an Excel spreadsheet for analysis.

**Figure 1:**
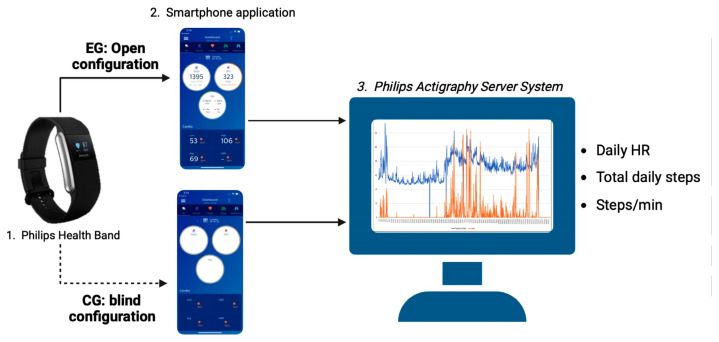
Schematic organization of the study protocol.

Training sessions were held from February to October 2022. Adherence to the training protocol was defined as the proportion of sessions completed during the entire study period. It was calculated only among patients with a complete W18 assessment, through PASS. Cardiorespiratory fitness, body composition, laboratory, and psychological data were measured at baseline (W0) and at the end (W18) of the study. Figure 1 shows the schematic organization of the study protocol.

### 2.3. Assessments

Cardiorespiratory fitness was tested during a 6-min walking test (6MWT) in a well-ventilated corridor at a temperature of 20–22 °C, between 8.30 a.m. and 12.00 p.m., at the same time of the day (±1 h) for W0 and W18. Participants were invited to arrive for the 6MWT at rest and well hydrated; to refrain from caffeine and alcohol during the 24 h before the test; and take their usual medication. 6MWT was performed according to the standardized guidelines of the American Thoracic Society [14]. Participants were instructed to walk as fast as possible for six minutes in a 30-m corridor, marked every 3 m, with turnaround points marked with a yellow cone. Every minute, participants were encouraged according to the standard protocol. During the test, $\dot{V}$O_2_, carbon dioxide production ($\dot{V}$CO_2_), the respiratory exchange ratio (RER), and pulmonary ventilation ($\dot{V}$E) were measured on a breath-by-breath basis by a portable telemetric metabolimeter (K5, Cosmed, Rome, Italy). Before the test, patients were fully familiarized with the testing procedures.

Body composition included height, body mass, body mass index (BMI), and percentage of fat mass (FM) and fat-free mass (FFM) by bioimpedentiometry (BIA 101 BIVA PRO, Akern srl, Pisa, Italy).

The laboratory parameters included the complete blood cell count, fasting total-, low-density lipoprotein (LDL), and high-density lipoprotein (HDL) cholesterol, triglycerides, glucose, insulin-glycated hemoglobin (HbA1c), creatinine, alanine transaminase (ALT), aspartate aminotransferase (AST), cluster of differentiation 4 (CD4+) and cluster of differentiation 8 (CD8+) T-cell counts, and HIV-1-RNA plasma level (Abbott Real Time HIV-1 assay). The Veterans Aging Cohort Study Risk (VACS) index, which estimates the risk of 5-year all-cause mortality in persons with HIV, was also evaluated [15].

The psychological parameters were assessed by validated Italian version questionnaires such as the Profile of Mood States (POMS) [16], Short Form Health Survey 36 (SF-36) [17], Global Physical Activity Questionnaire (GPAQ) [18], Fatigue Severity Scale (FSS) [19], and Activity Pacing Questionnaire 7 (APQ-7) [20].

### 2.4. Sample Size Calculation and Statistical Analysis

We expected a 67% proportion of adherent subjects to the exercise program in the EG (i.e., using the health watch and app), as observed in a previous study [9]. On the contrary, we expected a lower adherence, i.e., 40%, without digital supports (CG). Based on these preliminary data, a sample size of 40 patients per group (a total of 80 subjects) was estimated to be required in order to detect a 30% increase in the proportion of subjects with a ≥15% improvement in the baseline $\dot{V}$O_2_ at a steady state through 18 weeks of training, from 40% (hypothesized for CG, i.e., no use of the health watch and app) to 70% (hypothesized for the EG, i.e., with use of the health watch and app for tablet), with 80% power at an α of 0.05. However, based on feasibility recruitment issues (rate and duration), we here propose a pilot study with a total sample size of 24 participants (12 per arm).

Quantitative variables were expressed as the median and interquartile range (Q1–Q3). Due to the low sample size, nonparametric tests were used for analyses. The Wilcoxon matched-pairs signed-rank test was used to assess the changes between W0 and W18. The Fisher exact test was planned to be used to compare, between the two groups, the proportions of participants who improved the $\dot{V}$O_2_ at a steady state during 6MWT. The linear correlations between continuous variables were evaluated by the Spearman’s correlation coefficient test. The level of significance was set at 0.05. Graph Pad Prism Software, version 8.0, for Windows was used to perform statistical analysis (Graph Pad Software, San Diego, CA, USA).

## 3. Results

### 3.1. Study Population

**Figure 2:**
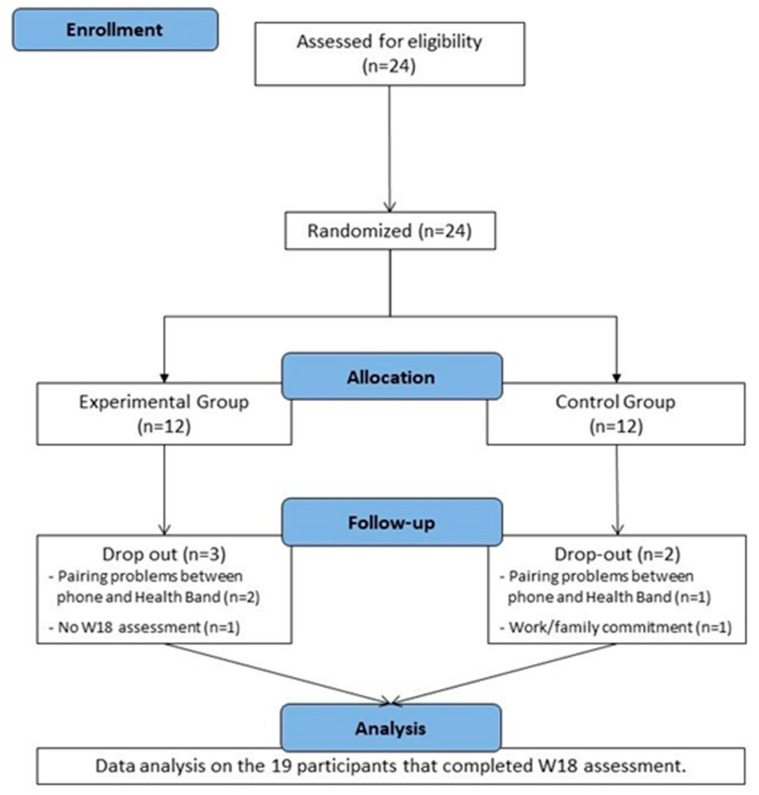
Participant flow diagram.

Twenty-four cART-treated subjects were screened; all were eligible to participate in the study and enrolled; 12 were allocated to the EG and 12 to the CG. However, three participants (EG = 2; CG = 1) never started the protocol activities because of pairing problems between the health-band and the smartphone. One participant in the EG did not present for the W18 assessment, and one participant in the CG dropped out during the training protocol because of work or family commitments. Nineteen participants completed the AP exercise program and W18 assessment, and we therefore considered them as a whole group for data analysis. Figure 2 shows participants disposition during the whole study.

The baseline characteristics of the 19 participants who completed the study are shown in Table 2.

### 3.2. Technical Issues with Wearable Devices and Training Monitoring

During the study, some main technical issues occurred with the use of the actigraphy devices, the smartphone app, and the PASS system.

Initially, we were able to start the program with only subjects who used smartphones with the iOS system (*n* = 6) because the devices using Android seemed not to support the app and therefore pair with the Health Band. The subsequent release of an app update (March 2022) enabled pairing for the 15 participants using the Android system. However, in three cases, the participant’s smartphone did not support the update. Finally, 21 out of 24 participants were able to download the app on their smartphones.

Once the app was downloaded, however, technical problems occurred for most EG participants with the download of the training data from the health band to the app. Therefore, the EG participants trained without the desired data feedback for most of the study period.

**Figure 3:**
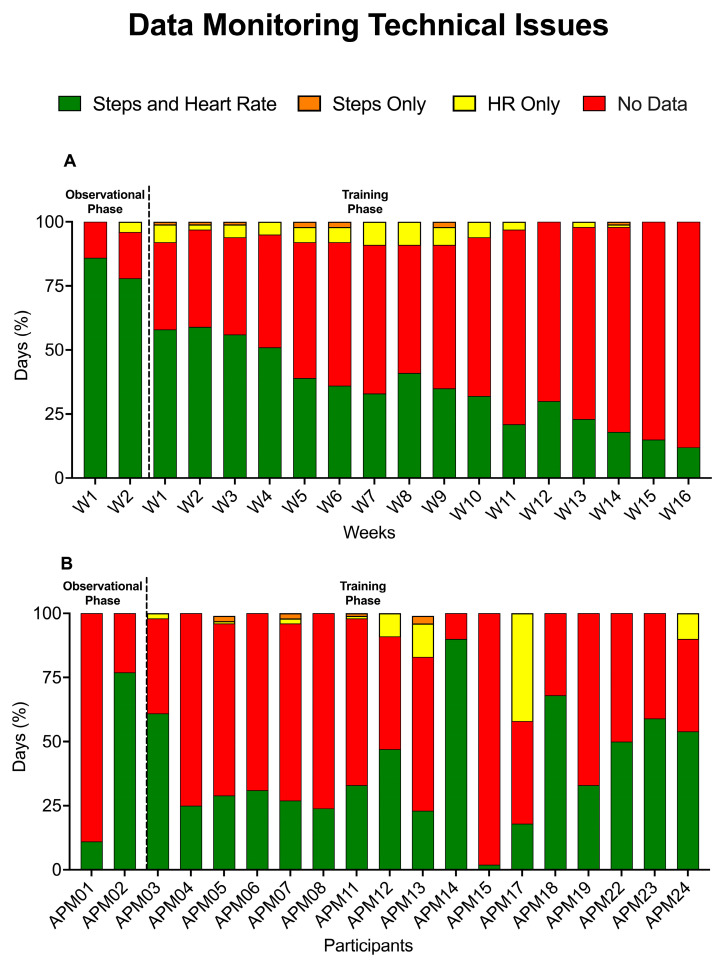
Summary of the information retrieved from the PASS system, including the proportion of days with information on the number of steps and/or HR for all 19 participants according to study week (**A**) and throughout the whole study period for each participant (**B**).

Another relevant issue was related to data synchronization from the app to the PASS (error message: “Synchronization with server failed”), which occurred with both iOS and Android systems. Consequently, coaches could not always receive the data reports and properly monitor physical activity. In several cases, the PASS reports were empty and/or contained only partial information, i.e., only data for HR or daily steps. Figure 3 summarizes the load of information that we could retrieve from the PASS system, including the proportion of days with information on the number of steps and/or HR for all 19 participants according to the study week (A) and throughout the whole study period for each participant (B). Considering that each participant was monitored for a total of 126 days, with a total of 2394 days in 19 participants, we were able to collect the complete monitoring of steps and heart rate in 963 out of 2394 days (40%). This proportion was significantly higher during the 2-week observational phase (219 out of 266, 83%) than in the training phase (744 out of 2128, 35%) (*p* < 0.0001, Chi-Square Test). The median percentage of days per patient with complete data monitoring was 33% (24–59) through the whole period, 100% (79–100) during the 2-week observational phase, and 25% (16–54) during the training phase. Overall, a complete report with >90% of days with complete data recording through the whole duration of the study was obtained in 1 participant; a proportion of >50% was available for 6 participants (31.6%) and of >25% for 13 participants (68.4%).

Because participants from the EG group were not able to train with the constant support of the data feedback due to the pitfalls of the digital instruments, there was in fact no EG group. Therefore, the findings at baseline and W18 and their changes over time could not be compared between the two groups and were evaluated altogether for both groups’ participants. In addition, adherence to the AP program could not be assessed due to a lack of complete training data transfer to the server.

### 3.3. Physical Activity Results

Overall, no significant changes at W18 compared to W0 were observed at the 6MWT for distance [504 (453–588) m vs. 522 (477–597) m, *p* = 0.859], absolute $\dot{V}$O_2_ [1.4 (1.2–1.7) L/min vs. 1.6 (1.4–2.0) L/min, *p* = 1.04], and relative $\dot{V}$O_2_ [15.5 (12.5–21.6) mL/kg/min vs. 18.1 (14.2–22.4) mL/kg/min, *p* = 0.389] at a steady state. An improvement from the baseline of 15% of steady-state $\dot{V}$O_2_ during 6MWT was observed in 6 out of 21 participants (32%). In addition, 6 out of 21 (32%) improved the walking distance in association with a median $\dot{V}$O_2_ increase of 53%. No significant correlations were observed between the percent change differences in the 6MWT distance compared to $\dot{V}$O_2_ changes at a steady state, nor for 6MWT distance or $\dot{V}$O_2_ percent changes compared to changes in all the other parameters examined.

### 3.4. Body Composition Results

Significant reductions were observed in body mass, BMI, and hip circumference in the whole group. No significant changes were observed for the other parameters (Table 3).

### 3.5. Laboratory Analysis Results

At W18, significant reductions were observed for total cholesterol and HDL-cholesterol, but not for the other laboratory variables (Table 4).

### 3.6. Psychological Profile Monitoring Results

No significant changes were observed at W18 compared to W0 for all the domains of the POMS questionnaire: depression [9 (8–13) A.U. vs. 8 (8–13) A.U., *p* = 0.441], fatigue [8 (6–9) A.U. vs. 7 (6–10) A.U., *p* = 0.634], tension [8 (6–12) A.U. vs. 8 (6–12) A.U., *p* = 0.872), vigor [17 (14–19) A.U. vs. 18 (15–19) A.U., *p* = 0.992], anger [8 (7–13) A.U. vs. 7 (7–10) A.U., *p* = 0.330], and total mood [19 (10–36) A.U. vs. 16 (12–27) A.U., *p* = 0.773)].

No significant changes were found at W18 compared to W0 for the SF-36 questionnaire items, about vitality [65 (55–80)% vs. 70 (50–80)% *p* = 0.433], physical functioning [100 (95–100)% vs. 95 (90–100)%, *p* = 0.073), bodily pain [100 (78–100)% vs. 90 (70–100)%, *p* = 0.534), general health perception [70 (45–85)% vs. 75 (50–85)%, *p* = 0.626], physical role functioning [100 (50–100)% vs. 100 (50–100)%, *p* = 0.500], emotional role functioning [100 (33–100)% vs. 67 (33–100)%, *p* = 0.625], social role functioning [88 (63–100)% vs. 75 (63–100)%, *p* = 0.999], and emotional wellbeing [76 (52–88)% vs. 72 (64–88)%, *p* = 0.999].

No significant changes at W18 were observed by the GPAQ questionnaire for total physical activity metabolic equivalent volume [4260 (1040–9189) mL O_2_/kg/min vs. 3120 (1440–5500) mL O_2_/kg/min, *p* = 0.922)] and minutes [885 (260–2033) minutes vs. 720 (330–1285) minutes, *p* = 0.982)], by the FSS questionnaire for perception of fatigue [3.5 (2.2–4.1) score vs. 3.3 (2.0–4.8) score, *p* = 0.925)], and by the APQ for engagement of pacing [2.0 (1.2–2.2) score vs. 1.8 (1.6–2.6) score, *p* = 0.347)] and perceived risk of over activity [1.5 (1.0–2.0) score vs. 1.0 (0.5–1.5) score, *p* = 0.168)].

## 4. Discussion

This pilot study aimed to explore the efficacy of smart technology and digital devices to support a 16-week exercise program of AP and monitor training data in sedentary persons with HIV.

Despite our previous encouraging experiences with the use of digital devices in favoring physical exercise [9], several technical issues encountered in this study significantly affected its implementation. On one hand, the technical problems prevented participants in the EG from exercising with the feedback support provided through the health band and mobile application. Consequently, they were not distinguished from the CG participants, and a comparison of data from the two groups, as per study protocol, was no longer possible. On the other hand, the failure to transfer data from the app to the server prevented the monitoring of training data, such as step counts and HR, with limitations to carrying out the AP program, making comparisons with other variables, and assessing adherence.

AP is a training strategy that allows the management of physical activity into feasible exercise portions [21]. Particularly, it includes specific activity planning and goal setting to increase exercise volume and intensity [22]. To this regard, we tailored an aerobic exercise prescription for each participant according to the data from the initial observational period (W0–W2), varying from 15 to 45 min of exercise volumes, with perceived intensities ranging from 0.7 to 3 of Borg CR10. The AP program was thought to be carried out by weighting step counts, as a measure of exercise volume, and HR, as a measure of intensity, against the perceived degree of fatigue, based on weekly telephone interviews. Because of the failure to download these data from most of the participants, the program was in fact based almost exclusively on the self-reported number and duration of the training sessions.

Exercise adherence is the most important issue that determines the success of a training protocol [11]. Therefore, we hypothesized that the combined use of a wearable device with a mobile application could improve exercise adherence by providing participants with feedback on their performance and thus increasing their motivation to exercise. Indeed, this hypothesis could not be proven because of the lack of an actual EG group. Also, actual adherence could not be assessed due to the failure to transfer training data to the server. However, a substantial improvement in the CRF, which was the primary objective of the study, was observed in only one-third of participants altogether, which suggests low adherence. It is likely that several variables reduced adherence. First, the training data were not available on the server in most of the cases; therefore, the AP program was monitored based on the self-reported volume and intensity of training by phone, which may not have been precise. Second, participants, who were initially keen to participate in the program, were giving in the end more attention to device functioning than to exercise activity, thus reducing their motivation to train properly. Third, problem-solving attempts, in collaboration with the technology provider experts, delayed the effective start of the program by weeks to months for most participants, which also likely contributed to reduced motivation.

Beyond being unable to assess the primary objective of the study and the overall low benefit of the program on CRF, we observed a substantial improvement in steady-state $\dot{V}$O_2_ and walking distance at 6MWT in one-third of the participants. It is likely that these participants were sufficiently motivated to exercise, irrespective of the functioning of the digital instruments. Also, improvements at W18 were observed for BMI, body mass, and blood lipids. This apparent dissociation between CRF and body composition and blood lipids had already been observed in other studies, where the percentage increase of CRF was not associated with percentage changes in body composition and blood lipids [4]. In contrast, although physical activity improves mood states and perceived health status in PLWH [23], we did not observe W18 changes in psychological parameters. It is also likely that the difficulties encountered by the participants might have affected the benefit of such a training program in terms of all the psychological outcomes.

Besides the technical issues encountered, additional limitations of this study were the low sample size and consequent low statistical power. Furthermore, women were underrepresented; indeed, only two women were enrolled and only one completed the study. However, as our study is the first to evaluate the use of digital devices with these specific features in a well-defined population and using an activity pacing approach, we designed a pilot study, which by definition, has a reduced sample size.

In any case, the information collected in this pilot study might be useful to the implementation of the use of digital devices for the prescription and monitoring of physical activity programs, both in research studies as well as in real life. To this regard, it is crucial to use only fully technically validated tools that are user-friendly and compatible among different brands. Indeed, the technical problems encountered in our study not only prevented the download of data and their feedback to participants, but also substantially reduced their motivation to exercise. It is important to consider that even minor technical issues might substantially impact on exercise programs. This may be particularly relevant in certain situations, as for instance with elderly people, who are often unfamiliar with new technologies, which may generate anxiety and subjective difficulties in performing. Beyond the technical issues, the AP program used in this study proved to be a feasible way to alternate and gradually increase activities and it was well received by the participants. The further development of AP-based programs for digital devices deserves consideration in upcoming clinical studies.

## 5. Conclusions

In conclusion, the technical problems encountered with the digital devices used in this study had a significant impact on training procedures, monitoring, assessments, and, likely, participants’ engagement and adherence to the program activity. While these tools may be important in providing feedback and motivation for the practice of physical exercise, potential technical difficulties may limit the expected benefits of physical exercise programs.

## Figures and Tables

**Table 1:** Physical activity progression through all 16 weeks of training for both continuous and intermittent training. Legend. %HR_max_: maximal heart rate.

	Continuous Training			Intermittent Training		
Weeks	Volume	Intensity		Volume	Intensity	
	*Minutes*	*%HR_max_*	*Borg CR10*	*Minutes*	*%HR_max_*	*Borg CR10*
**1–4**	15–25	60–70%	1.0–1.5	15–20	70–75%	1.0–1.5
**5–8**	25–35	60–70%	1.0–1.5	20–25	70–75%	1.0–1.5
**9–12**	35–45	60–70%	1.0–1.5	25–30	75–80%	1.0–1.5
**13–16**	20–40	60–75%	1.0–2.0	15–20	80–85%	1.0–2.0

**Table 2:** Baseline characteristics of the subjects who completed the study. Variables are expressed either as the number of participants (%) or as the median (Q1–Q3). Legend. ^a^: Chronic treatment, with no changes during the training period or the 6 weeks before; BMI: Body Mass Index; VACS: Veterans Aging Cohort Study Risk Index (This index includes: (i) age; (ii) laboratory tests: white blood cell count, HIV-1 RNA, hemoglobin, platelets, AST, ALT, and creatinine; (iii) liver fibrosis (FIB-4): composed of AST, ALT, platelets, and age; (iv) impaired renal function (eGFR): composed of age, gender, race, and creatinine; (v) HCV status: if the patient ever had a positive antibody test or detectable virus prior to the study; NRTI: nucleoside reverse transcriptase inhibitors; NNRTI: non-nucleoside reverse transcriptase inhibitors.

	All (*n* = 19)	EG (*n* = 9)	CG (*n* = 10)
**Demographic and general characteristics**			
Male (*n*, %)	18 (95%)	9 (100%)	9 (90%)
Age (years, median, Q1–Q3)	50 (39–61)	48 (34–61)	53 (45–61)
BMI (kg/m^2^, median, Q1–Q3)	28 (25–32)	30 (26–33)	26 (24–30)
Smokers (*n*, %)	5 (26%)	0 (0%)	5 (50%)
**Risk Group**			
Ex-intravenous drug users (*n*, %)	1 (5%)	0 (0%)	1 (10)
Men-having-Sex-with-Men (*n*, %)	13 (68%)	8 (89%)	5 (50%)
Heterosexual Infection (*n*, %)	3 (16%)	1 (11%)	2 (20%)
Unknown (*n*, %)	2 (11%)	0 (0%)	2 (20%)
**HIV Infection Variables**			
Nadir CD4^+^ (T-cells/μL)	472 (414–703)	559 (443–805)	386 (78–602)
Current CD4^+^ (T-cells/μL)	822 (698–980)	869 (746–914)	775 (650–1046)
Viral Load (<40 c/mL)	19 (100%)	9 (100%)	10 (100%)
VACS Index (median, Q1–Q3)	18 (5–26)	12 (0–33)	12 (0–26)
**Treatments** ^a^			
NRTI + integrase inhibitor (*n*, %)	15 (79%)	7 (78%)	8 (80%)
NRTI + NNRTI (*n*, %)	2 (11%)	1 (11%)	1 (10%)
NRTI + protease inhibitor (*n*, %)	1 (0.1%)	0 (0%)	1 (1%)
NNRTI + integrase inhibitor (*n*, %)	1 (0.1%)	1 (11%)	0 (0%)
Beta blockers (*n*, %) ^a^	2 (11%)	1 (11%)	1 (10%)
Other anti-hypertensive drugs (*n*, %) ^a^	6 (32%)	2 (22%)	4 (40%)
Statins (*n*, %) ^a^	2 (11%)	0 (0%)	2 (2%)
Fibrates (*n*, %) ^a^	1 (0.1%)	0 (0%)	1 (10%)

**Table 3:** Body composition changes at baseline (W0) and after 18 weeks of training (W18). Values are expressed as the median (Q1–Q3). W18 values were compared to W0 values by the Wilcoxson matched-pairs signed-rank test. BMI: body mass index.

	All Participants (*n* = 19)		
Parameters	W0	W18	*p*
Body mass (kg)	85.3 (73.3–101.2)	83.2 (72.8–99.3)	0.038
BMI (kg/m^2^)	27.7 (24.7–31.0)	27.7 (24.5–30.1)	0.040
Waist circumference (cm)	97.5 (93.0–109.0)	94.0 (86.0–104.0)	0.177
Hip circumference (cm)	106.0 (94.0–110.0)	104.0 (93.0–107.0)	0.027
Waist-to-hip ratio	0.9 (0.9–1.0)	1.0 (0.9–1.0)	0.963
Fat free mass (%)	75.8 (70.8–78.4)	75.8 (70.3–80.6)	0.984
Fat mass (%)	23.5 (20.2–29.2)	24.2 (19.4–29.7)	0.595

**Table 4:** Laboratory values at baseline (W0) and after 18 weeks of training (W18). Values are expressed as the median (Q1–Q3). W18 values were compared to W0 values by the Wilcoxson matched-pairs signed-rank test.

	All Participants (*n* = 19)		
Parameters	W0	W18	*p*
Red blood cells (K/μL)	5.0 (4.5–5.2)	4.9 (4.6–5.2)	0.083
White blood cells (K/μL)	6.2 (4.7–7.0)	6.3 (5.0–6.9)	0.374
Platet (K/μL)	202 (179–243)	199 (171–258)	0.126
Hemoglobin (mg/dL)	15.4 (14.5–16.2)	15.5 (14.1–15.8)	0.259
Total cholesterol (mg/dL)	188 (170–223)	186 (160–201)	0.049
LDL-cholesterol (mg/dL)	108 (93–145)	114 (98–133)	0.844
HDL-cholesterol (mg/dL)	45 (38–54)	41 (36–51)	0.045
Triglycerides (mg/dL)	137 (73–160)	116 (77–195)	0.616
Glucose (mg/dL)	78 (75–90)	82 (70–92)	0.446
Insulin (mg/dL)	14.0 (8.2–23.7)	14.6 (7.5–46.5)	0.346
Homa-index	3.0 (2.0–5.0)	3.0 (2.0–14.0)	0.127
HbA1c (%)	35.0 (33.0–37.0)	34.0 (32.0–38.0)	0.582
AST (U/L)	27.0 (21.0–42.0)	25.0 (22.0–32.0)	0.423
ALT (U/L)	32.0 (19.0–49.0)	28.0 (22.0–47.0)	0.448
Creatinin (mg/dL)	1.2 (1.0–1.2)	1.1 (1.0–1.2)	0.378
CD4 + T-cells/µL	857 (755–967)	851 (686–1000)	0.588
CD8 + T-cells/µL	792 (608–1260)	804 (712–1156)	0.826
VACS-index	12 (0–26)	12 (0–28)	0.125

## Data Availability

The data that support the findings of this study are available from the corresponding author upon reasonable request.
